# EXACKTE^2^: Exploiting the clinical consultation as a knowledge transfer and exchange environment: a study protocol

**DOI:** 10.1186/1748-5908-4-14

**Published:** 2009-03-13

**Authors:** France Légaré, Moira Stewart, Dominick Frosch, Jeremy Grimshaw, Michel Labrecque, Martine Magnan, Mathieu Ouimet, Michel Rousseau, Dawn Stacey, Trudy van der Weijden, Glyn Elwyn

**Affiliations:** 1 Research Center of the Centre Hospitalier Universitaire de Québec, Québec, Canada; 2 Department of Family and Emergency Medicine, Université Laval, Québec, Canada; 3 Department of Family Medicine, University of Western Ontario, London, Canada; 4 Department of Medicine, University of California, Los Angeles, USA; 5 Ottawa Health Research Institute, Ottawa, Canada; 6 Department of Medicine, University of Ottawa, Ottawa, Canada; 7 Department of Political Science, Université Laval, Québec, Canada; 8 Faculty of Health Sciences, School of Nursing, University of Ottawa, Ottawa, Canada; 9 Department of General Practice, School of Public Health and Primary Care (Caphri), Maastricht University, Maastricht, The Netherlands; 10 Department of Primary Care and Public Health, School of Medicine, Cardiff University, Cardiff, CF14 4YS, UK

## Abstract

**Background:**

While the evidence suggests that the way physicians provide information to patients is crucial in helping patients decide upon a course of action, the field of knowledge translation and exchange (KTE) is silent about how the physician and the patient influence each other during clinical interactions and decision-making. Consequently, based on a novel relationship-centered model, EXACKTE^2 ^(EXploiting the clinicAl Consultation as a Knowledge Transfer and Exchange Environment), this study proposes to assess how patients and physicians influence each other in consultations.

**Methods:**

We will employ a cross-sectional study design involving 300 pairs of patients and family physicians from two primary care practice-based research networks. The consultation between patient and physician will be audio-taped and transcribed. Following the consultation, patients and physicians will complete a set of questionnaires based on the EXACKTE^2 ^model. All questionnaires will be similar for patients and physicians. These questionnaires will assess the key concepts of our proposed model based on the essential elements of shared decision-making (SDM): definition and explanation of problem; presentation of options; discussion of pros and cons; clarification of patient values and preferences; discussion of patient ability and self-efficacy; presentation of doctor knowledge and recommendation; and checking and clarifying understanding. Patients will be contacted by phone two weeks later and asked to complete questionnaires on decisional regret and quality of life. The analysis will be conducted to compare the key concepts in the EXACKTE^2 ^model between patients and physicians. It will also allow the assessment of how patients and physicians influence each other in consultations.

**Discussion:**

Our proposed model, EXACKTE^2^, is aimed at advancing the science of KTE based on a relationship process when decision-making has to take place. It fosters a new KTE paradigm by putting forward a relationship-centered perspective and has the potential to reveal unknown mechanisms that underline effective KTE in clinical contexts. This will result in better understanding of the mechanisms that may promote a new generation of knowledge transfer strategies.

## Background

Many industrialized countries are facing new health care challenges, including expanded availability of health information [1], the extended role of patients in clinical decision-making [2], management of expectations regarding new treatments and technologies [3], and patient safety [4]. These challenges reinforce the need for changing the way we study knowledge translation and exchange (KTE) in clinical practice. We argue that, in clinical settings, the implementation of new evidence depends on two interdependent processes: the work of knowledge generation, distillation, and dissemination, and the exchange of information between physicians and patients, where evidence is used to enact clinical decisions. Thus, both physicians and patients have to share information, be sensitive to each other's preferences, arrive at a common understanding of each other's views and, ideally, come to an agreement on implementation of tests and treatments. In other words, the ideal model for KTE might be the sharing of decisions between a physician and a patient, a process that has the potential to be embedded in a specific relationship, known as 'shared decision-making' (SDM). By promoting the effective use of evidence in clinical practice, SDM could prove to be a valuable model for improving population health outcomes. Indeed, interventions aimed at fostering involvement of patients in clinical decisions were shown to reduce overuse of options not clearly associated with benefits for all (*e.g*., prostate cancer screening) [5] and enhance use of options clearly associated with benefits for the vast majority (*e.g*., cardiovascular risk factor management) [6]. Moreover, a recent review of the impact of SDM on patients' outcomes showed that in the context of a chronic illness, and when the intervention contains more than one session, SDM can be an effective method of reaching a treatment agreement [7]. Consequently, an ideal KTE model based on SDM would refer to the 'interactions between physicians and patients which result in mutual learning through the process of planning, producing, disseminating, and applying existing or new evidence in clinical decision-making.'[8].

However, conceptualization and operationalization of KTE as a relationship process between physicians and patients has important consequences for advancing the science of KTE and more specifically, the knowledge base of effective KTE interventions. Gaps in knowledge remain and include: lack of consensus on which aspects should be jointly considered; paucity of relationship-centered measures [9]; and inadequacy of analytical methods (*i.e*., failure to take into account the clustering of patients under physicians) [10]. Moreover, KTE research has failed to examine how the physician and the patient influence each other during the consultation. Until recently, assumptions arising from the 'two-communities theory' have caused patients and physicians to be studied as if living in separate worlds [11-13]. We argue that this phenomenon has hampered the development of effective KTE interventions in clinical settings, slowing the uptake of new evidence by the very actors whom that evidence most stands to benefit.

### EXACKTE^2^: conceptual model underlying this project

**Figure 1:**
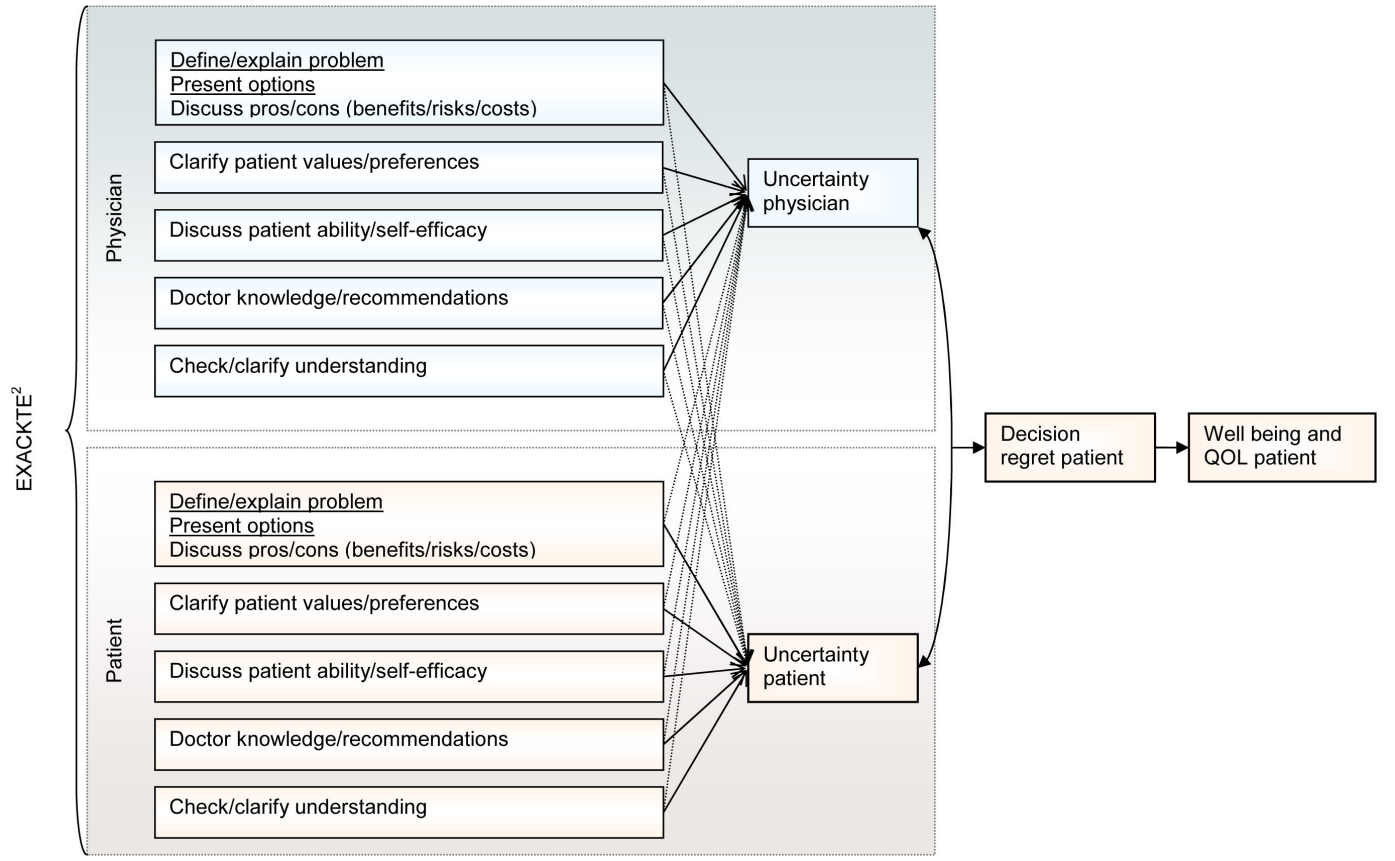
**EXACKTE^2 ^(Exploiting the Clinical Consultation as a Knowledge Transfer and Exchange Environment) model**.

**Figure 2:**
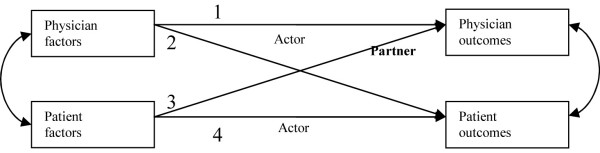
**Generic Actor-Partner Interdependence analytical Method (APIM) for physician and patient**.

We have proposed a novel relationship-centered model, EXACKTE^2 ^(EXploiting the clinicAl Consultation as a Knowledge Transfer and Exchange Environment) (Figure 1), where we foresee the consultation as an opportunity to exploit dyadic interaction, embedded in ongoing physician-patient relationships. It operationalizes the expected relationship phenomena between physicians and patients in consultations dealing with KTE using the essential elements of SDM and the analytical approach of the actor-partner interdependence model (APIM) (Figure 2) [14,15]. Relationship phenomena can be defined as phenomena 'pertaining to interpersonal dynamics that are more than the summation of the characteristics of the individuals interacting with each other.'[16]. In other words, based on EXACKTE^2^, the physician-patient interaction is an interpersonal system in which those involved relate to each other and not only to themselves.

We drew upon the systematic review of SDM by Makoul and colleagues to identify which of the essential elements would have an impact on the uncertainty levels of the physician and the patient [14]. The essential elements thus retained were: definition and explanation of the problem; presentation of the options; discussion of the pros and cons (*i.e*., the benefits, risks, and costs); clarification of patient values and preferences; discussion of patient ability and self-efficacy to act upon his or her treatment; presentation of doctor knowledge and recommendation; and checking and clarifying understanding. At an initial stage, these components lead to a specific level of personal uncertainty about a course of action on the part of both parties [13,17,18]. Then, as hypothesized by Falzer, it is when physicians and patients share their understanding not only of what is known but also of what is not known (what is scientifically and/or personally uncertain) that the parties find common ground [19]. Their level of agreement on their respective level of personal uncertainty will eventually affect decisional regret in patient and ultimately, this will possibly influence his or her well-being and quality of life (QOL). In other words, EXACKTE^2 ^conceptualizes the interpersonal transactions between physicians and patients as 'a meeting ground of unequal agents, with each party having a distinctive expertise and in which quality lies in responsiveness to uncertainty (scientific and personal) and where the shared decision promotes quality of care by facilitating this responsiveness' [19].

In the first stage of its application, EXACKTE^2 ^will allow us to assess: the influence of the essential elements of SDM assessed on the physician part on the physician's level of uncertainty (the actor effect) as well as on the patient's level of uncertainty (the partner effect) while controlling for the influence of the essential elements of SDM assessed on the part of the patient; and the influence of the essential elements of SDM assessed on the patient part on the patient's level of uncertainty (the actor effect)as well as on the physician's level of uncertainty (the partner effect) while controlling for the influence of the essential elements of SDM assessed on the part of the physician (Figure 1). In the second stage, EXACKTE^2 ^will allow us to assess the degree to which agreement between the physician's level of personal uncertainty and the patient's level of personal uncertainty impacts on the patient's decisional regret. In the final stage, EXACKTE^2 ^will allow us to assess the influence of the patient's decisional regret on his or her well-being and QOL.

EXACKTE^2^, our proposed model, has limitations. It does not pretend to describe nor explain the use of 'best evidence' (*e.g*., clinical practice guideline recommendations) by the clinical-patient dyad. Consequently, it does not focus on a 'best decision' that needs to be transferred in the consultation and hence, assessed. EXACKTE^2 ^fits the 'grey zone' of decision-making where most primary care health decisions occur [20]. These contexts are characterized either by scientific evidence that points to the need to balance harms and benefits within or between options, a concept known in shared decision-making as equipoise [21], or by the absence or insufficiency of scientific evidence. Moreover, EXACKTE^2^assumes that probabilities of risks and benefits in a population cannot be directly attributed at the individual level, and so uncertainty inevitably exists when considering individual decisions occurring in consultations. Consequently, this project addresses the need to reconcile KTE efforts with the need to determine ways by which a physician and a patient can be jointly supported to arrive at shared decisions.

### Study objectives

Our goal is to explore how patients and physicians influence each other during consultations where there is a need to transfer, exchange, and integrate knowledge on the part of both physicians and patients for clinical decisions to be made. Specific objectives are as follows:

1. Use EXACKTE^2^, a relationship-centered model, to identify which aspects should be jointly considered by physicians and patients in clinical interactions.

2. Provide further evidence on the validity and reliability of an identified set of existing relationship-centered measures based on objective one.

3. Assess the relationship phenomena between physicians and patients in clinical consultations dealing using dyadic analysis methods.

4. Assess the influence of the agreement between the physician's and the patient's uncertainty on the decisional regret and QOL of the patient.

Put simply, this research project emphasizes that 'the exchange, synthesis, and ethically-sound application of knowledge occurs within a complex system of interactions' in which the interactions are considered to be collaborative and two-way and thus, relationship-centered [11].

## Methods

### Clinical context

Primary care is the level of health care that: acts as the patient's gateway into the healthcare system for all of their health-related problems and needs; provides care focused on the individual and their context (patient-oriented instead of just disease-oriented); offers care for all but the most uncommon or unusual conditions; ensures continuity of care; and monitors the coordination or integration of care provided at other levels of the system or by other professionals [22]. Encompassing the widest possible spectrum of health conditions, primary care is by definition the forum where the greatest diversity of medical decisions takes place. For example, in a study that assessed for 903 consultations how comfortable family physicians and their patients were regarding a decision that had been made (*i.e*., personal uncertainty), 43% dealt with treatment decisions, 27% with diagnostic and screening tests, 12% with follow up and continuity of care, 6% with lifestyle issues, 5% with work-related issues, 4% with birth control, and 2% with vaccination [23]. Furthermore, on average, 90% of all monthly healthcare interactions occur in ambulatory clinical settings and 10% occur in hospital-based outpatient settings [24]. Together, these results emphasize that it is important to study decision-making in primary health care contexts because of the potential benefit to patient outcomes and ultimately to population health [14].

### Study design

We will use a cross-sectional study design in which immediately following a consultation between a recruited patient/physician pair, the parties will be asked to complete a set of relationship-centered questionnaires. We will also collect data about patients' outcomes at two weeks after the consultation. See Table 1 for timing and questionnaires type. These questions use the SDM model to assess the KTE interactions that took place between the physician and the patient during the course of their encounter. At two weeks, a research assistant will contact patients by telephone to administer two short questionnaires, one on decisional regret and one on the patient's QOL.

**Table 1:** Measurements and variables assessed

Type of variable	Variables assessed	Scale or sub-scale	Measures, Author, Year	Number of items	Timeframe		
					Entry	After consultation	2 weeks after consultation

1.0 Relationship-centered measures administered in physicians and patients							

1.1 Essential elements of SDM (Makoul & Clayman, 2006)							

	1.1.1 Knowledge-related Component						

	Define/explain problem	Information Giving	Medical Communication Competence Scale, Cegala 1998	9		x	
							
	Present options						
							
	Discuss pros/cons						

	Doctor knowledge/recommendations	Doctor recommendations	Patient-Physician Discordance Scale, Sewitch, 2001	7		x	
		
		Perception of the effectiveness of the decision	Decisional Conflict Scale, O'Connor 2005	7		x	

	Check/clarify understanding	Information verifying	Medical Communication Competence Scale, Cegala 1998	4		x	
		
		Feeling uninformed	Decisional Conflict Scale, O'Connor 2005	4		x	

	1.1.2 Value-related Component						

	Patient values/preferences	Values clarification	Decisional Conflict Scale, O'Connor 2005	4		x	
		
		Support	Decisional Conflict Scale, O'Connor 2005	3		x	
		
		Uncertainty	Decisional Conflict Scale, O'Connor 2005	3		x	

	Discuss patient ability/self-efficacy	Self-efficacy	Theory of planned behavior	3		x	

1.2 Other	1.2.1 Dyadic OPTION	Dyadic OPTION	Dyadic OPTION, Elwyn 2008	12		x	

2.0 Patient outcomes							

	2.1 Decisional regret	Decisional regret scale	Decisional Regret Scale, Brehault 2003	5			x (patient)

	2.2 Quality of life	Quality of life	Short-form 12, Ware 1996	12			x (patient)

3.0 Characteristics of physicians and patients							

	3.1 Attitude towards clinical decisions	Anxiety due to uncertainty	Physician's reaction to uncertainty scales, Gerrity 1995	5	x (physician)		
		
		Reluctance to disclose uncertainty to patients	Physician's reaction to uncertainty scales, Gerrity 1995	5	x (physician)		

	3.2 Sociodemographics (physicians and patients)				x (physician)	x (patient)	

4.0 Other							

	4.1 OPTION third observer	OPTION third observer	OPTION third observer, Elwyn 2001	12		x	

### Study population and recruitment strategy

French-speaking pairs of patients and physicians will be recruited in a practice-based research network (PBRN) located in Quebec City, Quebec. This PBRN is funded by the Canadian Foundation for Innovation. The network is composed of five family practice teaching units (FPTU) with about 50 to 60 physicians working at each site, including residents. Over 100,000 medical visits are carried out in total per year. English-speaking pairs will be principally recruited through the Family medicine Education and Research Network (FERN) of the Thames Valley Family Practice Research Unit (TVFPRU) in London, Ontario. About 200 family physicians belong to FERN, and TVFPRU has a list of 1,100 family physicians who might also be interested in participating in the study.

### Data collection procedures

Pairs of physicians and patients will be recruited using a procedure that we have successfully used in the past [25]. We will begin by enrolling physicians, asking them to complete a consent form, a socio-demographic questionnaire, and the physicians' reactions to uncertainty scale (PRU) [26]. During participating physicians' appointment hours, a research assistant will recruit patients in the waiting room at a randomly pre-determined time. Patients will be recruited according to the following criteria: ≥18 years old, able to read French or English according to the recruitment site, able to provide informed consent, not suffering from an acute condition that requires immediate medical intervention (*i.e*., transfer to emergency department), and able to report on a decision that they have made with the physician. Given that the recruitment procedures will be independent of the family physician and the time of recruitment randomly selected, we aim to protect against selection bias. As only one patient per physician will be recruited, patients who have already participated in the study once with a physician will be excluded. The goal of recruitment is to find one eligible patient per physician.

Once the subjects have been recruited, participating physicians will audio-tape one consultation with their consenting patient by using a digital audio recorder. Following the consultation, eligible patients (patients that have experienced that a decision was made) and physicians will be independently asked to complete a set of relationship-centered questions that assess their interaction. Based on prior projects that have shown this information to be valuable [25,27], we will ask each patient to describe the decision (*i.e*., the index decision) they have made with the physician in their own words. Following the patient's description of their decision, the patient will answer the questionnaire referring to the index decision immediately after the consultation. Patients' socio-demographics will also be assessed. Once the patient has completed their questionnaire, the research assistant will enter the decision identified by the patient on the physician's post-consultation questionnaire. The research assistant will then give the physician the post-consultation questionnaire to complete. The physician will be blinded to the patient's questionnaire. All audiotapes will be transcribed.

### Variables and measures

Using five published systematic reviews of instruments relevant to SDM research, two of which were performed by team members [28-32], we identified several questionnaires that map the various dimensions of EXACKTE^2^. These questionnaires have the potential of unraveling the relationship phenomena that underlie effective KTE between physicians and patients and can be administered to both parties alike. The same 'uncertainty' subscale of the decisional conflict scale (DCS) [17], for example, can determine how comfortable either physicians or patients are with the decision made [25]. Our review identified six measures of physicians' perceptions of the decision-making process [31] that have corresponding patient versions [18,33-36]. All are standardized measures that have been pilot-tested with physician-patient pairs.

### Relationship-centered dependent variable

This study will use the 'uncertainty' subscale of the DCS [17]. This subscale is comprised of three items (Cronbach's alpha = 0.70) [17]. It will be administered to both physicians and patients.

### Relationship-centered explanatory variables

The definition and explanation of the problem, the presentation of the options, and the discussion of the pros and cons (*i.e*., benefits, risks, and costs) will be assessed with the 'information-giving' construct of the medical communication competence scale (MCCS). This construct is comprised of nine items (Cronbach's alpha = 0.86) [34]. It will be administered to both physicians and patients.

Presentation of the doctor's knowledge and recommendations will be assessed using an instrument derived from the work by Sewitch and colleagues on patient-physician interactions [10]. This instrument assesses physician-recommended interventions from both the physician and the patient perspective according to four binary yes or no indicators: the prescription of medication; the scheduling of a further appointment; the consultation of another healthcare professional; and the conduct of further medical investigation [10]. It will be administered to both physicians and patients. Presentation of the doctor's knowledge and recommendations will be also assessed using the 'perception that an ineffective decision has been made' subscale of the DCS which is comprised of four items (Cronbach's alpha = 0.70 in physicians and 0.65 in patients) [23]. It will be administered to both physicians and patients.

Checking and clarifying the patient's understanding will be assessed with the 'information verifying' construct of the MCCS, which is comprised of four items (Cronbach's alpha = 0.78) [34] and with the 'feeling uninformed' subscale of the DCS, comprised of three items (Cronbach's alpha = 0.71) [23]. Both measures will be administered to both physicians and patients.

Exploration of values and preferences will be assessed with the 'value clarification' subscale of the DCS which is comprised of three items (Cronbach's alpha = 0.72) [23].

Discussion of the patient's ability and self-efficacy to act upon their choice will be assessed with the 'perceived behavioral control' construct of the Theory of Planned Behavior, which is comprised of three items [37]. Perceived behavioral control is a measure of the amount of control the individual perceives he or she has over the behavior in question, and is referred to as a measure of self-efficacy. As stated by Makoul and Clayman, 'the rationale for incorporating a patient's efficacy expectation parallels the argument for discussing patient preferences and values: both provide important perspective regarding acceptability of the options at hand' [14]. Team members have extensive expertise in the use of this scale in both patients and healthcare professionals in the context of SDM studies [27,38].

### Patient outcomes

At two weeks, the decisional regret scale (DRS), a five-item scale, will be used with patients (Cronbach's alpha = 0.81 to 0.92). This scale correlates with decision satisfaction (r = -0.40 to -0.60) and overall rated QOL (r = -0.25 to -0.27) [39]. QOL in patients will be assessed using the SF-12^® ^Health Survey [40]. These two scales will be administered only to patients.

### Statistical analyses and sample size

#### Sample size

We will solicit the participation of 300 family physicians. For each physician recruited, we will solicit the participation of one of the physician's patients (300 distinct patients in all). This will give us a total of 300 unique physician-patient pairs. One-half of the pairs will be French-speaking and the other half will be English-speaking. Given that between five and ten data entries are needed per item within each instrument (the largest number of items for one instrument being 12), we estimate that 150 distinct pairs in both languages will constitute an adequate sample size for performing factorial analyses as well as the other planned validity and reliability analyses for each scale [41,42]. This sample size is consistent with what other researchers have found: 'absolute sample size is more important than the functions of sample size in determining stable solutions' [43]. Thus, a minimum sample size of 100 to 200 observations is suggested. With this sample size, each set of relationship-centered measures will comprise the number of data entries required to perform statistical analyses in either language. Also, inclusion of 150 subjects will allow us to detect correlations between any two variables of 0.16 or higher (in absolute value) with alpha = 0.05 and beta = 0.20.

### Statistical analyses

To further assess evidence of the validity and reliability of the relationship-centered measures, internal consistency, *i.e*., how consistently subjects' scores on a measurement tool can be generalized to the domain of items that could be asked [44], will be used to estimate the reliability of the measures. Cronbach's alpha will be computed independently for each of the four subgroups of subjects (French and English, physicians and patients) and then for the overall groups of physicians and of patients [45].

Construct validity will first be assessed by confirmatory factor analysis (CFA) for each scale. This statistical method will be used to test for unidimensionality in each one of the relationship-centered measures. Results will help us determine if the empirical factor structure corresponds to the hypothesized theoretical unidimensional factor structure of each relationship-centered measure. CFA will be conducted with AMOS software. Since we will be recruiting unique physician-patient pairs, there will be no need to take the clustering of patients under physicians into account. However, clustering of the clinic from which physician-patient pairs will be assessed by computing an intra-class correlation coefficient for each measured outcome.

Second, construct validity will also be assessed by correlating the relationship-centered measure scores with OPTION, a validated third observer instrument (Cronbach's alpha = 0.79) that assesses SDM (convergent validity) [46]. Based on audio ratings of the consultations, two assessors will independently rate the encounters using observer OPTION (inter-rater reliability k = 0.71). Our *a priori *hypothesis is that the relationship-centered measures will correlate with observer OPTION in the expected direction (*e.g*., for the measure of personal uncertainty, we should be able to observe a positive correlation). Author GE and colleagues recently developed a dyadic reported version of OPTION with six family physicians in Cardiff, Wales. Using the results of this analysis, our team will triangulate the measurements of the consultation process using observer OPTION and the patient-physician version of dyadic OPTION.

Third, construct validity will be further assessed with a 'known groups' approach in physicians [47]. At entry into the study, physicians will complete the 'anxiety due to uncertainty' subscale (five items, Cronbach's alpha = 0.86) and the 'reluctance to disclose uncertainty to patients' subscale (five items, Cronbach's alpha = 0.79) of the physicians' reactions to uncertainty scale (PRU) [26]. Briefly, the PRU covered four areas of physicians' reactions to uncertainty derived from interviews with physicians: anxiety due to uncertainty; concern about bad outcomes; reluctance to disclose uncertainty to patients; and reluctance to disclose mistakes to physicians. The PRU assesses physicians' predisposition (*i.e*., a trait) to the uncertainty that is inherent to patient care from all sources. Our *a priori *hypothesis is that the relationship-centered measures will differentiate physicians with high scores on the 'anxiety due to uncertainty' as well on the 'reluctance to disclose uncertainty to patients' subscales from physicians with low scores (*e.g*., personal uncertainty of physicians will be higher in physicians with high scores on the 'anxiety due to uncertainty' as well on the 'reluctance to disclose uncertainty to patients' subscales than in physicians with low scores).

The fourth way that we will assess construct validity is with a 'known groups' approach in patients [47]. Based on a systematic review of patients' opinions on SDM, our *a priori *hypothesis is that some of the relationship-centered measures will discriminate between patients with high levels of education and patients with low levels of education (*e.g*., patients with high levels of education will have higher scores on the self-efficacy scale than patients with low levels of education). It will also discriminate between older patients and younger patients (*e.g*., older patients will have lower scores on the self-efficacy scale than younger patients) [2]. All of these analyses will first be performed independently for the physicians and the patients and subsequently for all subjects. This will help us to determine whether the different validity indices are adequate for physicians and patients in the relationship-centered approach to KTE.

To compare the physician and patient responses to the relationship-centered measures, invariance of the structural factor will be employed to verify that the factorial structure of the constructs is the same for patients and physicians. The invariance of the factorial structure will be assessed with CFA [48]. In other words, we will assess and compare the number of items that load on the latent dimension as well as their loading value. We will assess possible item bias using the Mantel-Haenszel method [49]. We will also estimate and test differences in variance and correlational structure within and across pairs using structural equation modeling [15]. Finally, we plan to assess the equivalence of our tools between the French-speaking and English-speaking data that will be collected.

To assess the relationship phenomena between physicians and patients, the Actor Partner Interdependence Model (APIM) will serve as the analytical framework to assess the assumed relationship phenomena between physicians and patients as it takes into account the interdependence between observations without losing possibly valuable information about what each member contributes to the pair. Hence, statistical analysis will be performed by means of structural equation modeling (SEM) with a maximum likelihood estimator. The dependent variable (outcome) will be personal uncertainty about a course of action in both physicians and patients. The predictor variables will consist of the essential elements of SDM: definition and explanation of the problem; presentation of the options; discussion of the pros and cons (*i.e*., the benefits, risks, and costs); clarification of patient values and preferences; discussion of patients ability and self-efficacy to act upon his or her treatment; presentation of doctor knowledge and recommendation; and checking and clarifying understanding as assessed in both physicians and patients. An initial APIM model will be constructed that allows all paths (effects) to be 'free'. Then, a second model will be constructed whereby all similar actor and partner paths are set to be equal, thereby assessing the similarities of effects between physicians and patients. Measures of model fit to be calculated include the chi-square, the comparative fit index (CFI) and the root mean square error of approximation (RMSEA). A non-significant chi-square value, CFI ≥ 0.95 and a RMSEA value of ≤ 0.06 will indicate good model fit [50]. Statistical analyses will be performed using SPSS (version 17.0) and AMOS (version 6.0) software.

To assess the relationship between the agreement of physicians and patients on the uncertainty with patients' decisional regret, first, using the methods of dyadic analysis proposed by Kenny and colleagues, an agreement score for physician-patient pairs on the 'uncertainty' subscale will be computed [15]. This agreement score will be entered into a general linear regression model as an explanatory variable of the decisional regret assessed in patients at two weeks. The relationship between patients' decisional regret and patients' QOL will be assessed by regressing the physical and the mental health component scores of the SF-12 on patients' decisional regret scores.

### Ethical Considerations

Participants will be asked to complete consent forms. Ethics approval for the project was obtained from the Research Ethics Board of the Centre de Santé et de Services Sociaux de la Vieille Capitale in Quebec City, Canada (final approval 25 November 2008; ethics number #2008–2009-23). Physicians and patients will not be financially remunerated for their participation.

## Discussion

Our proposed model, EXACKTE^2^, addresses important knowledge gaps in KTE science. More specifically, EXACKTE^2 ^goes beyond the idea that knowledge needs only to be generated and disseminated in order for medical care to reflect new research findings and translate into decisions. It also challenges the conviction that new interventions oriented toward physicians and/or new interventions oriented toward patients will solve the current disconnection between the generation of evidence and its application at the point if care, that is, within consultations where dyads of physicians and patients are expected to share decisions. Instead, our model takes the innovative approach that for effective KTE to occur in primary care, we must first understand the interpersonal dynamics within the decision-making process that take place between physicians and patients.

The principal target audiences for our results comprise educators and the SDM and KTE research communities. We will also share our findings with organizations of health professionals, patient representative associations and healthcare policy makers interested in enhancing the quality of the clinical decision-making process, especially for Canadians facing 'grey-zone' decisions (*i.e*., health decisions occurring in contexts of scientific uncertainty) [20].

The four main deliverables of our project are: EXACKTE^2^, a new conceptual model of KTE based on the essential elements of SDM; further evidence of the validity and reliability of relationship-centered measures that fit with our proposed model; evidence of the presence or the absence of relationship phenomena in KTE interactions within consultations in primary care; and an improved knowledge base for elaborating future KTE interventions.

This study also has the potential to enhance the knowledge in some of the research areas identified as priorities by the Canadian Institutes of Health Research, Canada's premier health research agency. First, it will provide new hypotheses for future intervention studies aiming at translating evidence into clinical practices. Second, it will reinforce a patient-centered care approach that places high value on relationships [51]. In short, we hope that the evidence produced here will reveal new mechanisms that underlie effective KTE in clinical contexts, mechanisms that will promote a new generation of KTE strategies in turn.

## Competing interests

The authors declare that they have no competing interests.

## Authors' contributions

FL and GE developed the protocol and all authors contributed to the final version. FL is its guarantor.
